# The Conformational Control Inhibitor of Tyrosine Kinases DCC-2036 Is Effective for Imatinib-Resistant Cells Expressing T674I FIP1L1-PDGFRα

**DOI:** 10.1371/journal.pone.0073059

**Published:** 2013-08-29

**Authors:** Yingying Shen, Xiaoke Shi, Jingxuan Pan

**Affiliations:** Department of Pathophysiology, Zhongshan School of Medicine, Sun Yat-sen University, Guangzhou, People's Republic of China; McGill University, Canada

## Abstract

The cells expressing the T674I point mutant of FIP1-like-1-platelet-derived growth factor receptor alpha (FIP1L1-PDGFRα) in hypereosinophilics syndrome (HES) are resistant to imatinib and some second-generation tyrosine kinase inhibitors (TKIs). There is a desperate need to develop therapy to combat this acquired drug resistance. DCC-2036 has been synthesized as a third-generation TKI to combat especially the Bcr-Abl T315I mutant in chronic myeloid leukemia. This study evaluated the effect of DCC-2036 on FIP1L1-PDGFRα-positive cells, including the wild type (WT) and the T674I mutant. The *in vitro* effects of DCC-2036 on the PDGFRα signal pathways, proliferation, cell cycling and apoptosis of FIP1L1-PDGFRα-positive cells were investigated, and a nude mouse xenograft model was employed to assess the *in vivo* antitumor activity. We found that DCC-2036 decreased the phosphorylated levels of PDGFRα and its downstream targets without apparent effects on total protein levels. DCC-2036 inhibited proliferation, and induced apoptosis with MEK-dependent up-regulation of the pro-apoptotic protein Bim in FIP1L1-PDGFRα-positive cells. DCC-2036 also exhibited *in vivo* antineoplastic activity against cells with T674I FIP1L1-PDGFRα. In summary, FIP1L1-PDGFRα-positive cells are sensitive to DCC-2036 regardless of their sensitivity to imatinib. DCC-2036 may be a potential compound to treat imatinib-resistant HES.

## Introduction

Idiopathic hypereosinophilic syndrome (HES) refers to the pathological presence of more than 1.5×10^9^/L of nonreactive eosinophils in peripheral blood lasting for more than 6 months with organ involvement [1]. According to WHO classification system, HES can be classified as molecularly characterized eosinophilic disorders, such as platelet-derived growth factor receptor (PDGFR)-rearranged myeloid neoplasm, eosinophilia associated with phenotypically abnormal and/or clonal T lymphocytes, and chronic eosinophilic leukemia (CEL) [2]. Several genetic abnormalities such as PDGFRα, PDGFRβ or FGFR1 have been observed in PDGFR-rearranged myeloid neoplasm [3], and the FIP1L1-PDGFRα fusion gene generated by the interstitial deletion on chromosome 4q12 has been reported to account for 5% to 15% [4]. The expression of FIP1L1-PDGFRα can promote activation of pro-survival signal pathways, such as extracellular signal-regulated kinases (Erk), signal transducers and activators of transcription (STAT), JNK and PI3K-Akt signaling in CD34+ hematopoietic progenitor cells [5], [6].

FIP1L1-PDGFRα is sensitive to imatinib treatment and patients with HES can be successfully treated with imatinib (100 mg/day) [7]. However, the secondary mutation T674I FIP1L1-PDGFRα in its kinase domain has been found in imatinib-refractory HES or CEL. T674I FIP1L1-PDGFRα, analogous to T315I Bcr-Abl in CML, is also resistant to the second-generation TKIs, such as nilotinib [8]. Novel agents for imatinib-resistant HES are needed.

DCC-2036, a conformational control inhibitor of ABL1, showed remarkable efficacy in murine bone marrow transplantation model of Bcr-Abl^T315I^ CML [9] and potently suppressed the T315I Bcr-Abl in primary patient cells *in vitro* and *in vivo* [10]. Besides, DCC-2036 exhibited high selective activity for FLT3, TIE2 and SRC-family kinases [10]. Given the significant effect of DCC-2036 on Bcr-Abl^T315I^ CML, we herein evaluated the efficacy of DCC-2036 against the FIP1L1-PDGFRα-expressing cells, including EOL-1 cell line and BaF3 cell lines harboring the WT or T674I FIP1L1-PDGFRα, to examine DCC-2036 as a strategy to overcome the drug-resistance of HES.

## Materials and Methods

### Reagents

DCC-2036 was purchased from Selleck (Houston, TX, USA), sorafenib and imatinib were from Alexis Biochemicals (Plymouth Meeting, PA). They were dissolved in dimethyl sulfoxide (DMSO) at a final concentration of 20 mmol/L (mM) and stored in aliquots at−20°C. Antibodies against PARP, Bcl-2, X-linked inhibitor of apoptosis protein (XIAP) and cytochrome *c* (clone 6H2.B4) were obtained from BD Biosciences Pharmingen (San Jose, CA, USA); antibodies against phospho-PDGFRα (Y1018), phospho-Erk1/2 (T202/Y204), Erk 1/2, phospho-Akt (S473), total Akt, Bax, caspase-3, phospho-Bim (S69) and the MEK inhibitor U0126 were purchased from Cell Signaling Technology (Beverly, MA); antibodies against phospho-STAT3 (Y705), total STAT3, total PDGFRα were products of Upstate Technology; antibodies against Mcl-1 (S19), apoptosis-inducing factor (AIF), and Bax were from Santa Cruz Biotechnology (Santa Cruz, CA, USA); antibodies against Bim were obtained from Stressgen Bioreagents (Columbia, Canada); antibodies against Survivin were purchased from Novus Biotechnology (Littleton, CO, USA); cycloheximide (CHX) and antibodies against Actin, active-caspase3 were from Sigma-Aldrich (St. Louis, MO); the PI3K inhibitor LY294002 and MG132 was bought from Calbiochem (San Diego, CA); anti-rabbit immunoglobulin G horseradish peroxidase-conjugated and anti-mouse immunoglobulin G antibodies were obtained from Pierce Biotechnology (Los Angeles, CA, USA) [11], [12]; the plasmid Bim-EL was from Origene (Rockville, MD, USA); His-ubiquitin (His-Ub) plasmid was obtained from Abcam (Cambridge, MA); Ni–nitrilotriacetic acid (NTA) agarose beads were purchased from Invitrogen (Carlsbad, CA).

### Cell culture

EOL-1 cell line harboring the FIP1L1-PDGFRα fusion oncogene and BaF3 cells carrying WT or T674I FIP1L1-PDGFRα (BaF3-WT or BaF3-T674I) were described previously [13], [14]. EOL-1 cells and BaF3 cells were cultured in RPMI-1640 medium (Invitrogen, Guangzhou, China), added with 10% fetal calf serum (Carlsbad, CA). Cells were cultured at 37°C and in water vapor-saturated air with 5% CO_2_.

### Cell viability assay

Viable cells were quantified by MTS assay (Cell Titer 96 Aqueous One Solution reagent; Promega, Madison, WI) as previously described [11], [12]. 100 μL BaF3 cells or EOL-1 cells were plated in triplicate in 96-well plates (2×10^4^ cells per well) and then cultured with various concentrations of DCC-2036 for 72 hours. Twenty μL MTS solution per well was added 4 hours before culture termination. The absorbance was read on a 96-well plate reader at wavelength 490 nm. The drug concentration resulting in 50% decrease in the number of live cells (IC_50_) was determined.

### Immunoblotting analysis

Cells were incubated with different concentrations of DCC-2036 for indicated durations, washed, and then harvested by preparing whole lysates in RIPA buffer (1×PBS, 0.5% sodium deoxycholate, 1% NP-40, 0.1% sodium dodecyl sulfate) supplemented with freshly added 10 mM β-glycerophosphare, 10 mM NaF, 1 mM phenylmethylsulfonyl fluoride (PMSF), 1 mM sodium orthovanadate, and 1×Roche complete Mini protease inhibitor cocktail) (Roche, Indianapolis, IN, USA) [12], [15]. As for AIF and cytochrome *c* release detection, the cytosolic fraction was prepared in digitonin extraction buffer [1 mM PIPES (pH 6.8), 300 mM sucrose, 0.015% (wt/vol) digitonin, 3 mM MgCl_2_, 5 mM EDTA, 100 mM NaCl, and 1 mM PMSF] [12], [15], [16]. The concentration of protein was measured in a modified Lowry method at 750 nm wavelength (protein assay, Bio-Rad Laboratories, Hercules, CA). Proteins were resolved by molecular size by sodium dodecyl sulfate (SDS)-polyacrylamide gel electrophoresis (PAGE) and transferred to nitrocellulose membranes by electroblotting. Proteins were probed with the corresponding antibodies described above and chemiluminescence was used to detect the change of protein level with standard immunoblotting procedures according to the Manufacturer's instruction [17].

### Apoptosis assay by flow cytometry

After DCC-2036 treatment, cells were collected, washed with 1× Binding buffer, and then stained for about 20 minutes with Annexin V-FITC (fluorescein isothiocyanate) for EOL-1 cells or Annexin V-PE (phycoerythrin) for BaF3 cells expressing WT or T674I FIP1L1-PDGFRα. Propidium iodide (PI) or 7-amino-actinomycin (7-AAD) was added before flow cytometric analysis depending on the fluorophore attached to Annexin V. Apoptosis was assessed with the FACS Calibur flow cytometer and CellQuest Pro software (Becton Dickinson) as previously described [11], [12].

### Cell-cycle analysis by flow cytometry

After drug treatment, cells were harvested, fixed in 75% alcohol, and stained with propidium iodide (PI; Sigma-Aldrich) for 1 hour in dark according to manufacturer's guildance. Cell-cycle distribution was analyzed with FACS Calibur flow cytometer with CellQuest Pro software as described [11], [12].

### Clonogenicity assay

BaF3-WT and BaF3-T674I cells (2×10^5^/mL) were exposed to indicated concentrations of DCC-2036 (DMSO for control) for 36 hours, then cells were collected, washed with 1× PBS, then cultured in methylcellulose media (Cat#03231; Stem Cell Technology). Absent of drug administration, after 7 days incubation at 37°C, colonies with more than 50 cells were counted under an inverted phase-contrast microscope. The capability of colony formation was assessed.

### siRNA transfection

The siRNA against human Bim and non-targeting siRNAs (Mock siRNA as control) were purchased from Dharmacon RNA technology (Blenheim, England). Bim siRNA or Mock siRNA was transfected into the EOL-1 cells; then cells were incubated with indicated concentrations of DCC-2036 for 24 hours. Cell death and related protein levels were detected. The Cell Line Nucleofector Kit T involved was from Amaxa technology (Guangzhou, China), and Program O-17 was chosen according to the manufacturer's instructions [18].

### In vivo ubiquitination assay

The *in vivo* ubiquitination assay was carried out as previously described [19], [20]. In brief, BaF3 cells expressing T674I FIP1L1-PDGFRα were transfected with the indicated plasmids for 24 hours and then treated with or without DCC-2036 at 400 nM for 8 hours. Then the cells were lysed by a denaturing buffer (6 M guanidine-HCl, 0.1 M Na_2_HPO_4_/NaH_2_PO_4_, 10 mM imidazole), followed by nickel bead purification and immunoblot analysis. The ubiquitinated BimEL was quantified by densitometry using Image J.

### Nude mouse xenograft model

Nude *nu/nu* BALB/c mice (Slaccas Company, Shanghai, China) were housed in barrier facilities at the animal center of Sun Yat-sen University, Guangzhou, China. The mice were bred in a 12-hour light-dark cycle environment with food and water available ad libitum. 9×10^6^ of BaF3 cells harboring the T674I FIP1L1-PDGFRα resuspended in Matrigel (Becton-Dickson Biosciences Pharmingen, San Jose, CA) were inoculated subcutaneously on the flank of the mice, which were about 4∼6 weeks old [21]. When tumors were palpable (∼100 mm^3^), the mice were divided at random into two groups. One group received vehicle as control (0.5% Carboxymethylcellulose/1% Tween-80), the other group received DCC-2036 (200 mg/kg, daily, oral gavage) dissolved in 0.5% Carboxymethylcellulose/1% Tween-80. The tumors were calculated with calipers every other day, and the tumor volumes were measured by the following formula: *a* ^2^×*b*×0.4, where *a* is the smallest diameter and *b* is the diameter perpendicular to *a*. Other indicators of general health, such as body weight, feeding behavior, and motor activity of each animal were also monitored. After administration of drug or vehicle for about two weeks, the mice were sacrificed, and the tumor xenografts were immediately dissected, weighted, stored and fixed.

### Ethics Statement

This study was conducted based on the recommendations in the Guide for the Care and Use of Laboratory Animals of the National Institutes of Health. Animal studies were approved by Institutional Animal Care and Use Committee of Sun Yat-sen University (Protocol Number: 2012–0403). Sodium pentobarbital anesthesia methods and other efforts were taken to minimize animal suffering.

### Immunohistochemical staining

Tumor xenografts were fixed in formalin, then embedded in paraffin and sectioned, and maxVision Kit (Maixin Bio) was used to immunostain the xenograft sections (4 μm). After incubation with the primary antibody of rabbit anti-Ki67 [21], each slide was applied with about 50 μL maxVision reagent, followed by 0.05% diaminobenzidine and 0.03% H_2_O_2_ in 50 mmol/L Tris-HCL (pH 7.6) to develop the color; then the slides were counterstained with hematoxylin. A negative control for Ki67 was developed for each xenograft specimen by replacing rabbit anti-Ki67 with pre-immune rabbit serum.

### Statistical analysis

All experiments were carried out at least three times, and data are expressed in the form of mean ± standard deviation (SD) unless otherwise stated. Statistical analysis was conducted with GraphPad Prism 5.0 software (GraphPad Software, San Diego, CA, USA). Two-tailed Student's *t* test was employed to compare the data between two groups, while One-Way ANOVA with *post hoc* intergroup comparison by Tukey test was for comparing more than two groups. A p<0.05 was considered statistically significant.

## Results

### DCC-2036 decreases the levels of phosphorylated-PDGFRα and its downstream signaling

**Figure 1:**
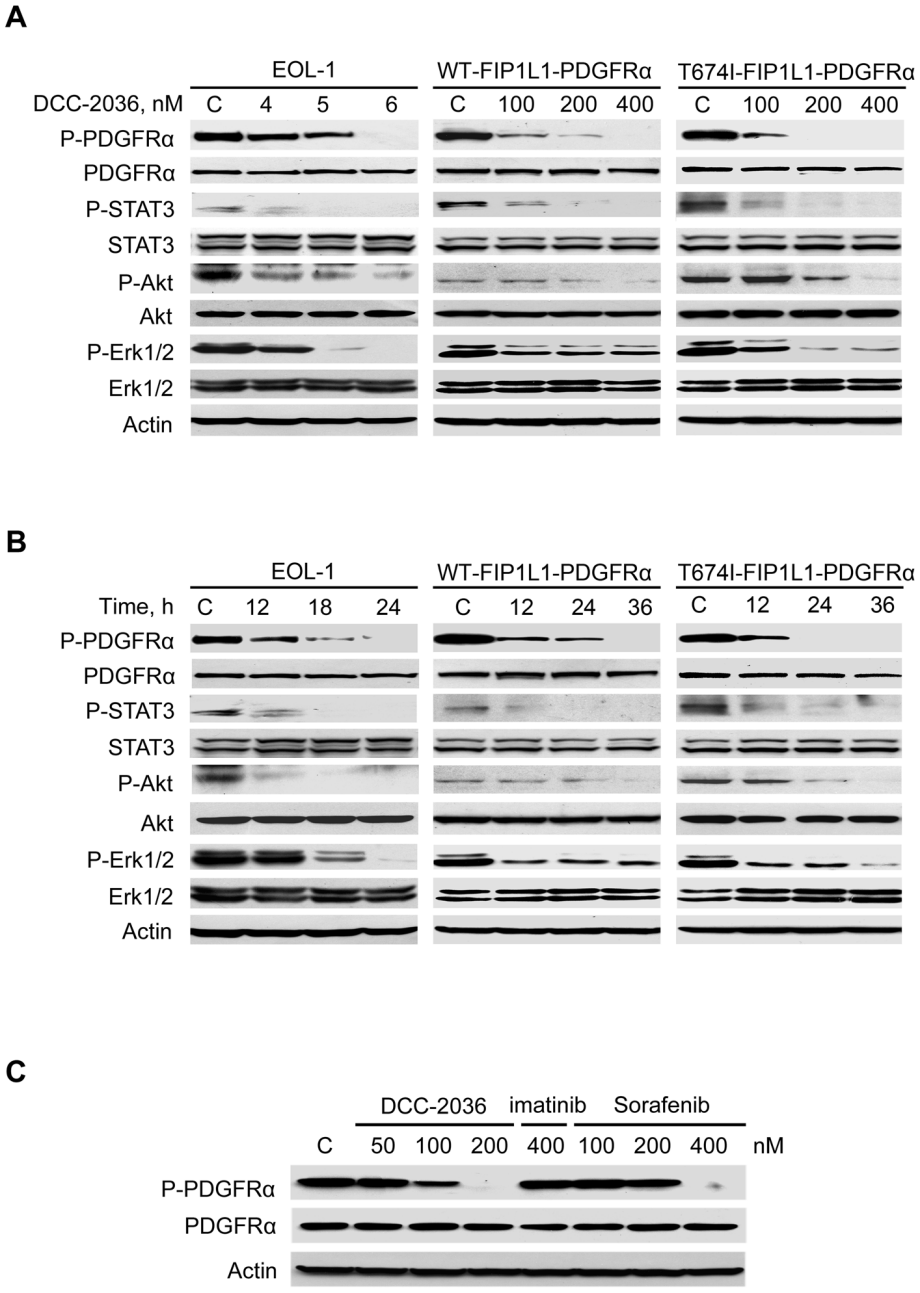
DCC-2036 inhibits the phosphorylated-PDGFRα level and its downstream targets. A, EOL-1 cells and BaF3 cells harboring WT or T674I FIP1L1-PDGFRα were incubated with indicated concentrations of DCC-2036 for 24 h (EOL-1) or 36 h (BaF3-WT and -T674I mutant), the phosphorylated and total levels of PDGFRα, STAT3, Akt and Erk1/2 were analyzed by immunoblotting. B, EOL-1 cells and BaF3 cells expressing WT or T674I FIP1L1-PDGFRα were treated with DCC-2036 for indicated durations with different concentrations (6 nM for EOL-1 cells, 400 nM for BaF3 cells), the phosphorylated and total levels of indicated proteins were analyzed by immunoblotting. C, BaF3-T674I cells were exposed to the indicated concentrations of DCC-2036, imatinib or sorafenib for 36 h, the phosphorylated PDGFRα and total PDGFRα were analyzed by immunoblotting. Imatinib was used for comparison.

EOL-1 cell line, derived from a patient with FIP1L1-PDGFRα-positive HES [14] and BaF3 cells expressing wild-type or T674I FIP1L1-PDGFRα were employed in the study. EOL-1 cells exhibited a higher sensitivity to DCC-2036. Exposure to 6 nM DCC-2036 for 24 hours completely abolished the phosphorylation of PDGFRα in EOL-1 cells. Because the FIP1L1-PDGFRα T674I mutant in HES mainly contributes to the imatinib-resistance [22], [23], we explored whether DCC-2036 could inhibit PDGFRα in FIP1L1-PDGFRα-positive cells regardless of the mutational status. DCC-2036 effectively decreased the phosphorylated-PDGFRα in BaF3 cells expressing FIP1L1-PDGFRα T674I mutant in a concentration- and time-dependent manner while the total protein level of PDGFRα was not affected (Figure 1A & B). Similar results were observed in BaF3 cells expressing WT FIP1L1-PDGFRα.

Next, we measured the phosphorylation of downstream signaling proteins of PDGFRα in the three cell lines. In concordance with our previous finding, the phosphorylated levels of downstream signaling molecules such as the STAT3, Erk1/2, and Akt were all decreased by DCC-2036, without apparent impact on total protein levels (Figure 1A & B). Therefore, DCC-2036 inhibited the activation of phosphorylated-PDGFRα and its downstream signaling molecules in FIP1L1-PDGFRα-positive cells despite of the mutational status of FIP1L1-PDGFRα.

Because tyrosine kinase inhibitor sorafenib was effective for imatinib-resistant FIP1L1-PDGFRα T674I mutation cells [23], we also compared the inhibitory effect of DCC-2036 with sorafenib in the BaF3-T674I cells. We found that DCC-2036 decreased the phosphorylated-PDGFRα with lower concentrations than sorafenib, indicating that DCC-2036 might be more potent (Figure 1C).

### DCC-2036 inhibits the proliferation of EOL-1 cells and BaF3 cells harboring wild-type or T674I FIP1L1-PDGFRα

**Figure 2:**
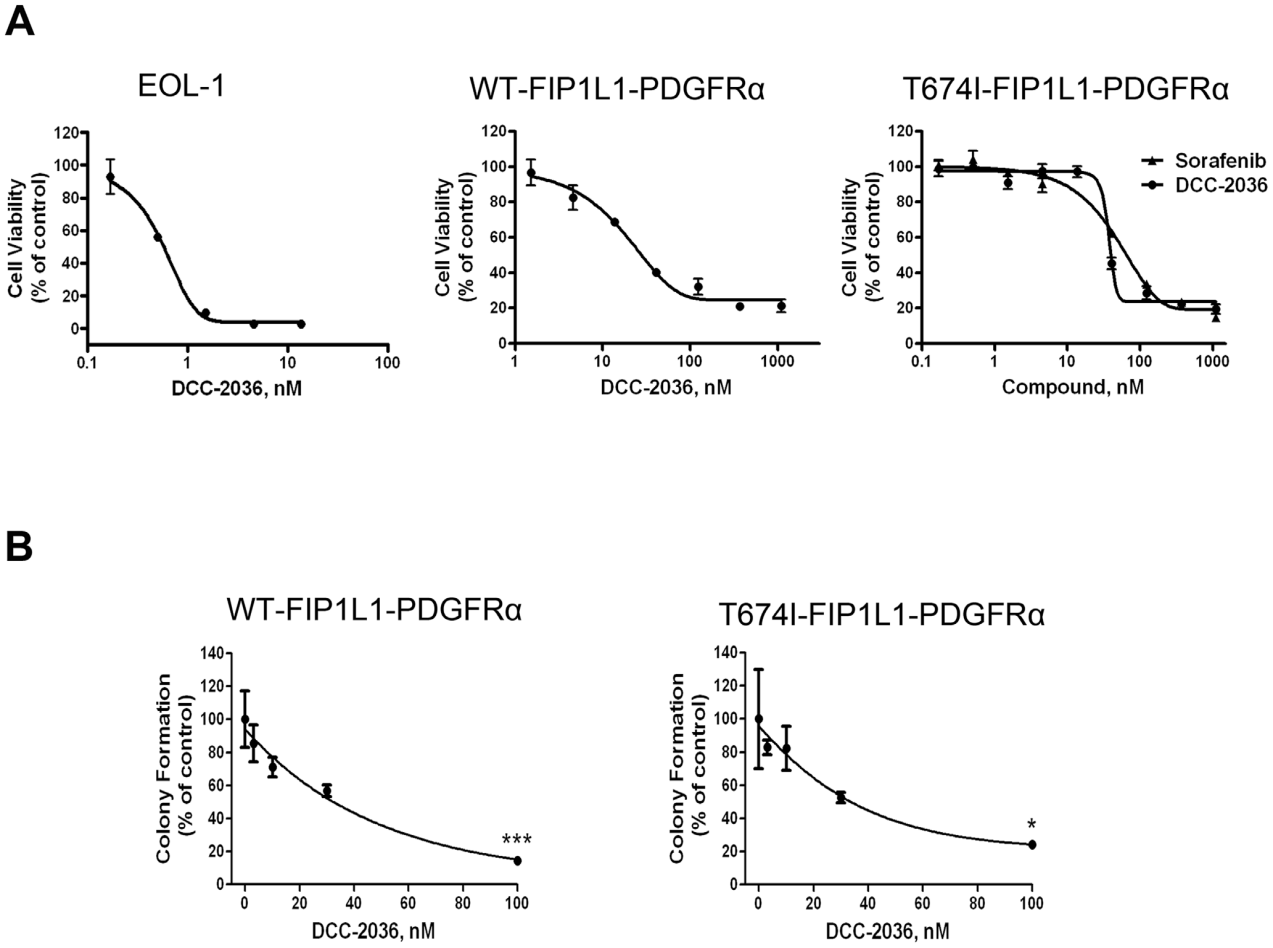
DCC-2036 inhibits the growth of FIP1L1-PDGFRα-positive cells. A, DCC-2036 inhibits the cell viability of FIP1L1-PDGFRα-positive cells. EOL-1, BaF3-WT, and BaF3-T674I cells were exposed to increased concentrations of DCC-2036 or sorafenib (a positive control) for 72 hours followed by the MTS assay. Data are from experiments in triplicate and expressed as mean ± standard deviation (SD). B, Clonogenicity of BaF3 cells harboring WT or T674I FIP1L1-PDGFRα in methylcellulose was inhibited by DCC-2036 in a concentration-dependent manner. One-way ANOVA with *post hoc* intergroup comparison with control by Tukey test. *P<0.05, ***P<0.0001. Data were mean ± standard error of the mean (SEM), and the experiments were performed in triplicate.

To evaluate the inhibitory effect of DCC-2036 on cell proliferation, EOL-1 cells and BaF3 cells were treated with different concentrations of DCC-2036 for 72 hours, and then cell viability was measured with the MTS assay. DCC-2036 potently decreased the cell viability of EOL-1, BaF3 cells expressing WT or T674I FIP1L1-PDGFRα (IC_50_ = 0.8 nM, 32.2 nM and 40.3 nM, respectively, Figure 2A). Sorafenib also decreased the cell viability of BaF3 cells expressing T674I FIP1L1-PDGFRα but with a slightly higher IC_50_ (IC_50_ = 89.0 nM, Figure 2A), which again suggested that might be more effective than sorafenib in the FIP1L1-PDGFRα T674I mutation cells. In addition, we carried out the colony-formation experiment to evaluate the effect of DCC-2036 on the anchorage-independent growth of BaF3 cell lines. BaF3 cells expressing WT or T674I FIP1L1-PDGFRα were co-cultured with indicated concentrations of DCC-2036 for 36 hours, then cells were seeded in methylcellulose without DCC-2036. The colony-forming capability was assessed according to the number of colonies. The colony-forming capabilities of both cell lines were inhibited by DCC-2036 in concentration-dependent manners (Figure 2B). Therefore, DCC-2036 actively suppressed the growth and proliferation of cells expressing WT or T674I FIP1L1-PDGFRα.

### DCC-2036 increases sub-G_1_ population in FIP1L1-PDGFRα-expressing cells

**Figure 3:**
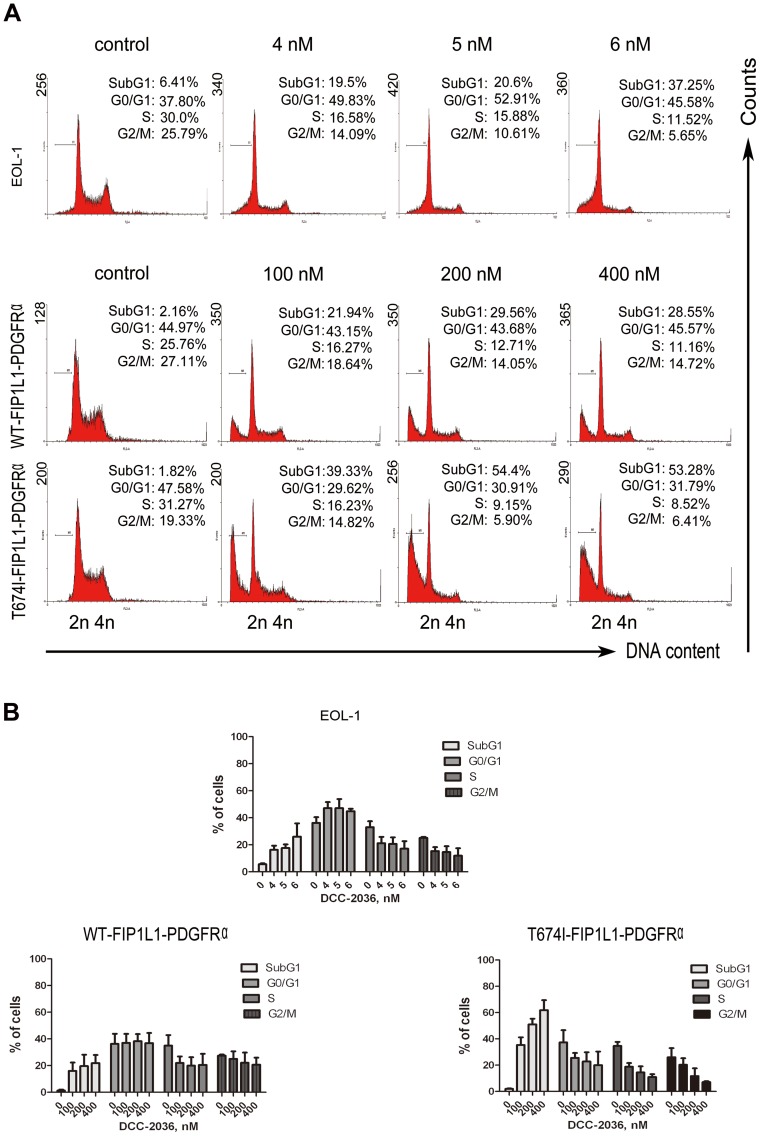
DCC-2036 increases sub-G_1_ population in FIP1L1-PDGFRα-positive cells. EOL-1 cells and BaF3 cells expressing WT or T674I FIP1L1-PDGFRα were treated with indicated concentrations of DCC-2036 (24 hours for EOL-1 cells, 36 hours for BaF3 cells); then cells were collected, washed, stained with propidium iodide and analyzed with flow cytometry. A) Representative graphs of three independent experiments; B) Statistical charts. Columns, mean; error bars, SD.

We next explored whether the cell cycle was affected by DCC-2036 administration. EOL-1, BaF3 cells expressing WT or T674I FIP1L1-PDGFRα were treated with indicated concentrations of DCC-2036 for different durations (24 h for EOL-1 cells and 36 h for BaF3 cells), and their DNA contents were analyzed with PI, a DNA-intercalating dye. All three cell lines had an increased sub-G_1_ apoptotic population but no apparent alteration in cell-cycle phase distribution compared with control (Figure 3).

### DCC-2036 results in apoptosis of cells expressing WT or T674I FIP1L1-PDGFRα

**Figure 4:**
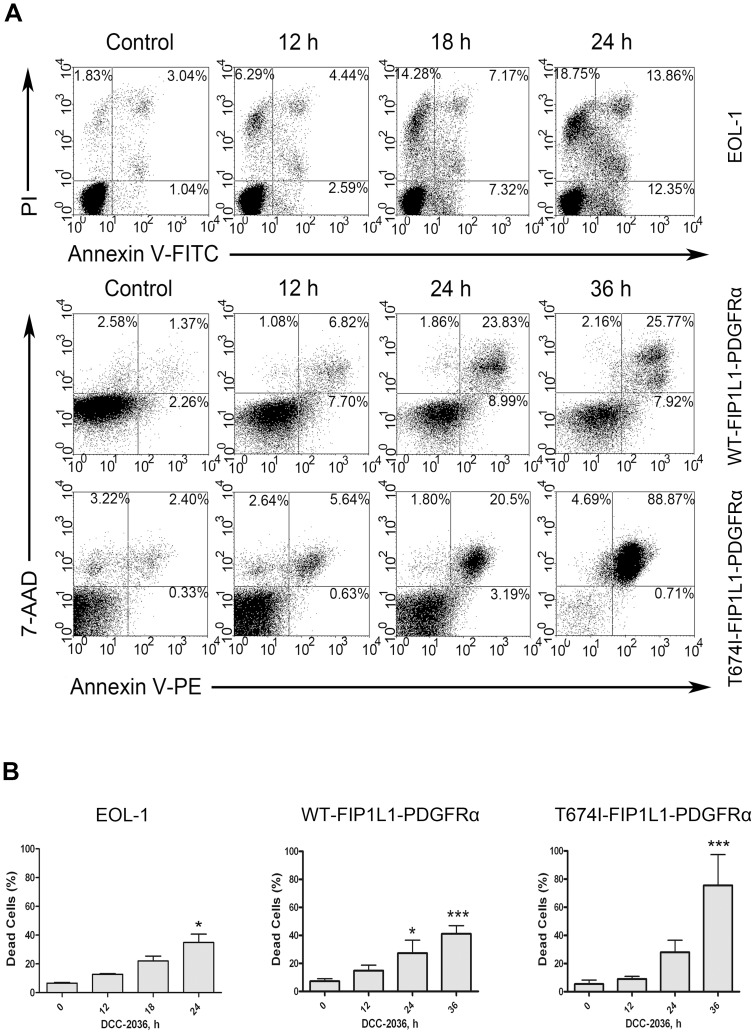
DCC-2036 induces apoptosis of FIP1L1-PDGFRα-expressing cells with flow cytometry assay. EOL-1 cells and BaF3 cells expressing WT or T674I FIP1L1-PDGFRα were cultured with DCC-2036 for indicated durations at 6 nM (EOL-1 cells) or 400 nM (BaF3 cells), and then cells were collected, washed, fixed and stained with Annexin V-FITC/PI (EOL-1) or Annexin V-PE/7-AAD (BaF3 cells) to detect the cell death with flow cytometry. A, representative of three independent experiments; B, statistical charts, One-way ANOVA with *post hoc* intergroup comparison with control by Tukey test. *P<0.05, ***P<0.0001. Data are expressed as mean ± SD.

To demonstrate the effect of DCC-2036 on apoptosis of FIP1L1-PDGFRα–expressing HES cells, cells were incubated with various concentrations of DCC-2036 for indicated durations (24 h for EOL-1 cells, 36 h for BaF3 cells), then stained with Annexin V/PI (EOL-1 cells) or Annexin V-PE/7-AAD (BaF3 cells) to assess cell apoptosis. DCC-2036 induced apoptosis in a time- and dose-dependent manner (Figure 4, Figure S1A and S1B). Treatment of 6 nM DCC-2036 for 24 hours led to ∼40% cell death of EOL-1 (Figure 4), while the apoptosis of BaF3 cells expressing WT or T674I FIP1L1-PDGFRα after treatment with 400 nmol/L DCC-2036 for 36 hours reached ∼40% and ∼80%, respectively, suggesting that BaF3-T674I cells were more sensitive to DCC-2036 than imatinib (Figure 4).

**Figure 5:**
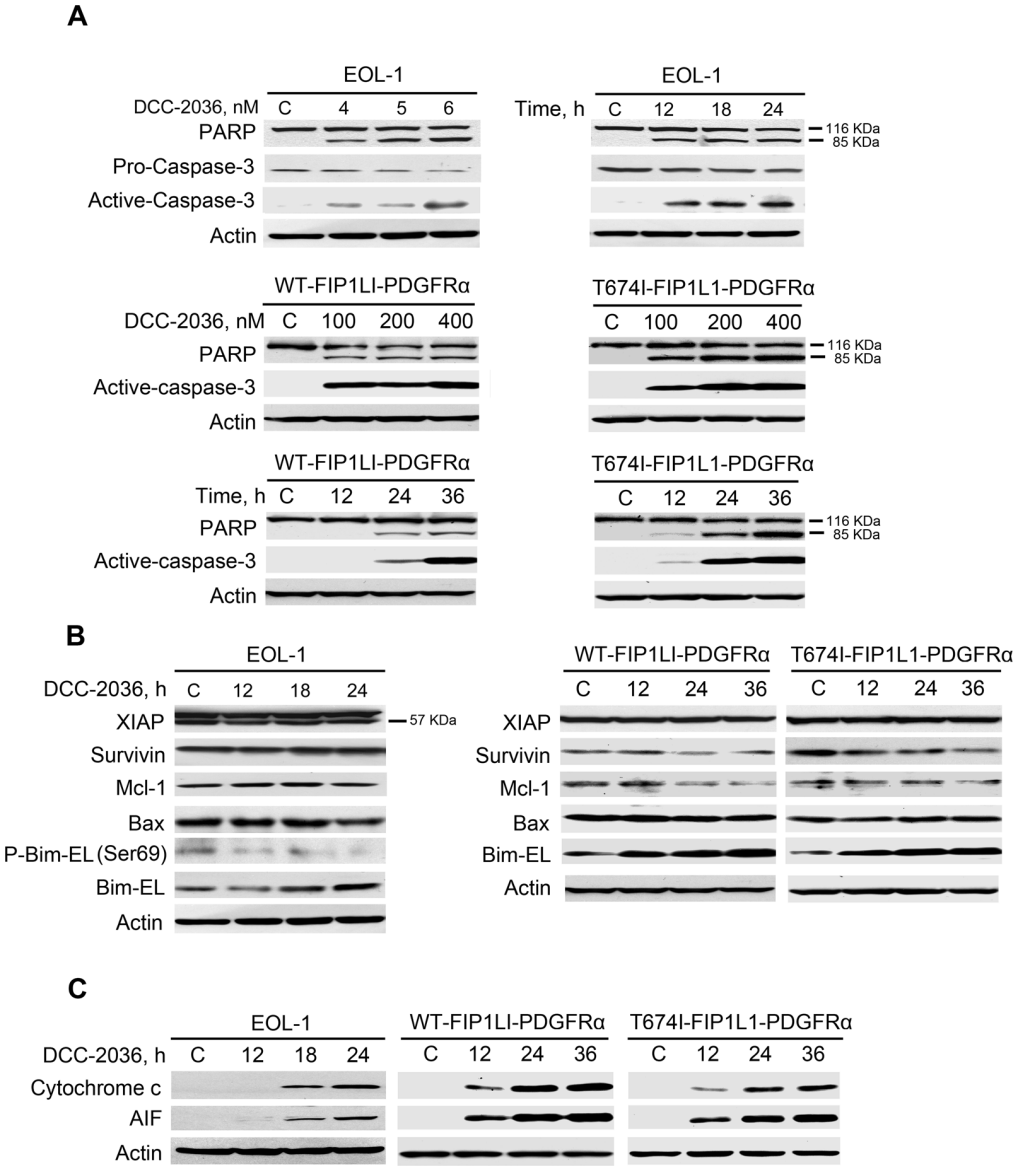
DCC-2036 induces apoptosis of FIP1L1-PDGFRα-expressing cells with Western blotting assay. A, immunoblotting of PARP cleavage and caspase-3 activation was shown in EOL-1 cells and BaF3 cells in a concentration- and time-dependent manner. B, Impact of DCC-2036 on apoptosis-related proteins. EOL-1 cells, BaF3-WT and BaF3-T674I cells were exposed to a fixed concentration of DCC-2036 (6 nM for EOL-1 cells, 400 nM for BaF3 cells) for indicated durations and then levels of apoptosis-related proteins were detected by Western blotting. C, the cytosolic AIF and cytochrome *c* were detected in EOL cells (6 nM) and BaF3 cells (400 nM) in a time- dependent manner by immunoblotting.

Immunoblotting revealed that the cleavage of PARP and caspase-3, hallmarks of apoptosis, was increased in a concentration- and time-dependent manner, and were concurrent with the decline of PARP (116 kDa) and pro-caspase-3 (32 kDa) (Figure 5A). We observed no apparent change in other apoptosis-related proteins such as XIAP, Survivin, Bax, and Mcl-1, but there was an overt increase in Bim-EL protein level in a time- and dose-dependent manner (Figure 5B and Figure S1C).

Moreover, the release of AIF and cytochrome *c* from mitochondria into the cytosol, has been considered an indicator of cell apoptosis in the early phase [15], [24], [25]. Western blot exhibited a substantial release of AIF and cytochrome *c* into cytosol in both EOL-1 cells and BaF3 cells expressing WT or T674I FIP1L1-PDGFRα time-dependently (Figure 5C). These data may infer that DCC-2036 induced apoptosis in HES cells through the intrinsic (mitochondrial) pathway.

### Elevated Bim-EL is essential for apoptosis induced by DCC-2036

**Figure 6:**
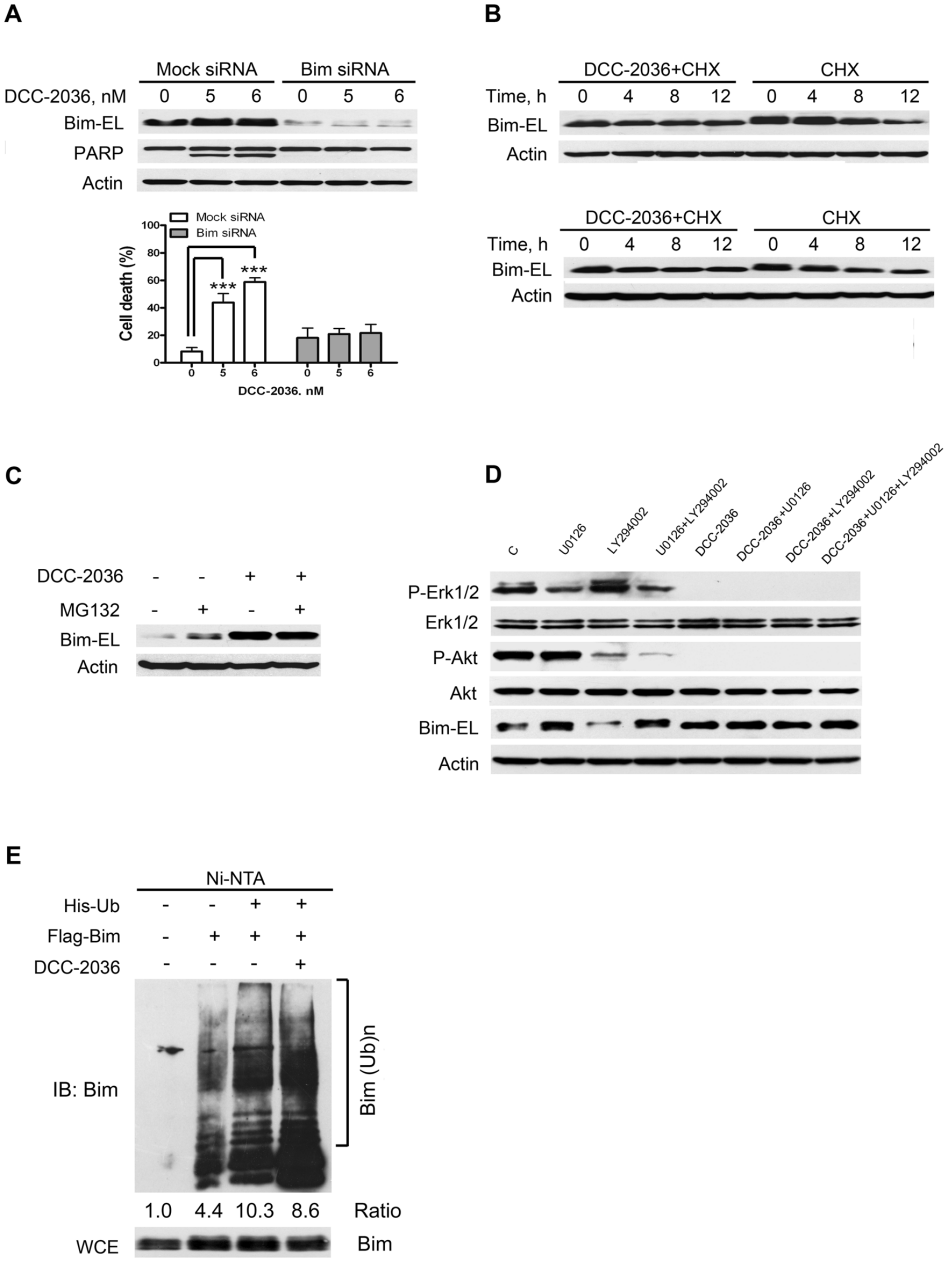
MEK-dependent up-regulation of Bim-EL confers the DCC-2036 induced apoptosis. A, knockdown of Bim attenuated the apoptosis in EOL-1 cells. EOL-1 cells were transfected with human Bim siRNA or non-specific control pool (Mock siRNA), respectively. Then cells were exposed to the indicated concentrations of DCC-2036. Twenty-four hours after treatments, the levels of indicated proteins were analyzed with immunoblotting (*top*). Cell death was detected with trypan blue in triplicate (*bottom*). Columns, mean; error bars, SD. One-way ANOVA with *post hoc* intergroup comparison with control by Tukey test. ***P<0.0001. B, DCC-2036 decelerated the turnover rate of Bim-EL protein. BaF3 cells expressing WT or T674I FIP1L1-PDGFRα were exposed to 400 nM DCC-2036 for 2 hours, and then cycloheximide (CHX) was added (100 μg/mL). Cells were harvested at the indicated time points and underwent the Western blotting. C, DCC-2036 elicited a proteosome-dependent degradation of Bim-EL protein. After a 2-hour pretreatment with MG132, BaF3-T674I cells were also treated with or without 400 nM DCC-2036 for 12 more hours. Then cells were harvested, and the levels of Bim-EL protein were analyzed by Western blotting. D, MEK-dependent upregulation in Bim-EL protein in DCC-2036 treated cells. EOL-1 cells were treated with a MEK inhibitor (U0126, 20 μM), a PI3K inhibitor (LY294002, 25 μM), DCC-2036 (6 nM) or combination of them, respectively, for 24 hours, then cells were harvested and the indicated proteins were analyzed by immunoblotting. E, DCC-2036 decreases Bim-EL polyubiquitination. BaF3-T674I cells were transfected with Bim-EL along with His-ubiquitin (His-Ub). After 24 hours, the cells were treated with or without DCC-2036 at 400 nM for 8 hours, and then collected for *in vivo* ubiquitination of Bim-EL. The ubiquitinated BimEL was quantified by densitometry using Image J. The scale of ubiquitinated BimEL were normalized to that of relevant total BimEL, and then normalized to control. The ratio was shown. Ni–nitrilotriacetic acid (NTA), nickel bead precipitate; WCE, whole-cell extracts.

It has been reported that Bim was critical for erlotinib-induced apoptosis in EGFR mutant lung cancer cells [26] and imatinib-induced apoptosis in KIT dependent GIST cells [27]. We investigated whether up-regulation of Bim (Figure 5B) was critical for DCC-2036-induced-apoptosis in HES cells. We transfected EOL-1 cells with Bim siRNA duplexes to silence the expression of Bim, or Mock siRNA as control, then EOL-1 cells were incubated with indicated concentrations of DCC-2036 for 24 hours. Apoptosis was evaluated by immunoblotting of PARP and by counting live cells with trypan blue dye exclusion. As shown in Figure 6A, knockdown of Bim remarkably attenuated apoptosis mediated by DCC-2036 in EOL cells, suggesting that Bim might be a critical mediator in DCC-2036-induced apoptosis in HES. Of note, Bim-extra long (EL) but not Bim-long (L) or Bim-short (S) is the primary isoform detected in these cells [28], we thus turned our attention to Bim-EL isoform in subsequent experiments.

### DCC-2036 mediates Bim-EL up-regulation by inhibiting its degradation

Given the critical role of Bim-EL, we further explored the potential mechanism that DCC-2036 induced Bim-EL up-regulation. The mRNA level of Bim showed no significant change by qRT-PCR (data not shown), which suggested that DCC-2036 might increase Bim-EL at posttranscriptional levels. We examined whether the turnover rate of Bim-EL protein was affected by DCC-2036. BaF3 cells harboring WT or T674I FIP1L1-PDGFRα were treated with or without 400 nM DCC-2036 for 2 hours, then 100 μg/mL cycloheximide (CHX), a translation inihibitor, was added, and then cells were harvested at the indicated time point, immunoblotting showed that the turnover of Bim-EL was decelerated compared with cells absent of DCC-2036 treatment (Figure 6B), suggesting that DCC-2036 may increase Bim-EL protein level by reducing its turnover rate.

Next, we investigated whether the proteasome pathway was involved in the degradation of Bim-EL protein. BaF3-T674I cells were pre-treated with 1 μM MG132 for 2 hours, then incubated with 400 nM DCC-2036 for 12 hours. Western blotting analysis revealed that the proteasome inhibitor MG132 led to increased expression of Bim-EL and there was no difference in the Bim-EL protein level between the MG132 treatment alone and combination of MG132 and DCC-2036 (Figure 6C), indicating that the up-regulation of Bim-EL depended on the proteasome pathway.

It has been reported that Bim could be regulated by MEK-ERK or PI3K-Akt pathway [29], [30], [31]. Our data showed that both pathways were inhibited by DCC-2036, so we further explored which pathway was predominantly responsible for up-regulation of Bim-EL mediated by DCC-2036. EOL-1 cells were exposed to U0126 (a MEK inhibitor), LY294002 (a PI3K inhibitor), DCC-2036 or combination of them respectively. Western blot analysis revealed that DCC-2036 or U0126 alone induced up-regulation of Bim-EL. In contrast, EOL-1 cells treated with LY294002 did not exhibit any remarkable increase in Bim-EL protein level. Furthermore, combination of the two or three compounds didn't display additive effects on up-regulation of Bim-EL compared with U0126 treatment alone. All these data supported that DCC-2036 increased Bim-EL protein level predominantly through MEK-ERK pathway (Figure 6D).

Erk1/2 can phosphorylate Bim-EL at serine69 (Ser69) eliciting a rapid polyubiquitination and proteosomal degradation [31]. In our study, we observed the protein level of phospho-Bim-EL (Ser69) declined significantly with DCC-2036 treatment of EOL-1 cells (Figure 5B and Figure S1C), which was consistent with declined phospho-Erk1/2 resulted from decreased phospho-PDGFRα by DCC-2036 (Figure 1A and 1B). Moreover, we found that polyubiquitinated Bim-EL decreased with treatment of DCC-2036 for 8 hours by ubiquitination assay in intact cells (Figure 6E), which suggested that the total Bim-EL in cells was increased.

### DCC-2036 suppresses the growth of xenografted BaF3 cells harboring FIP1L1-PDGFRα T674I mutant in nude mice

**Figure 7:**
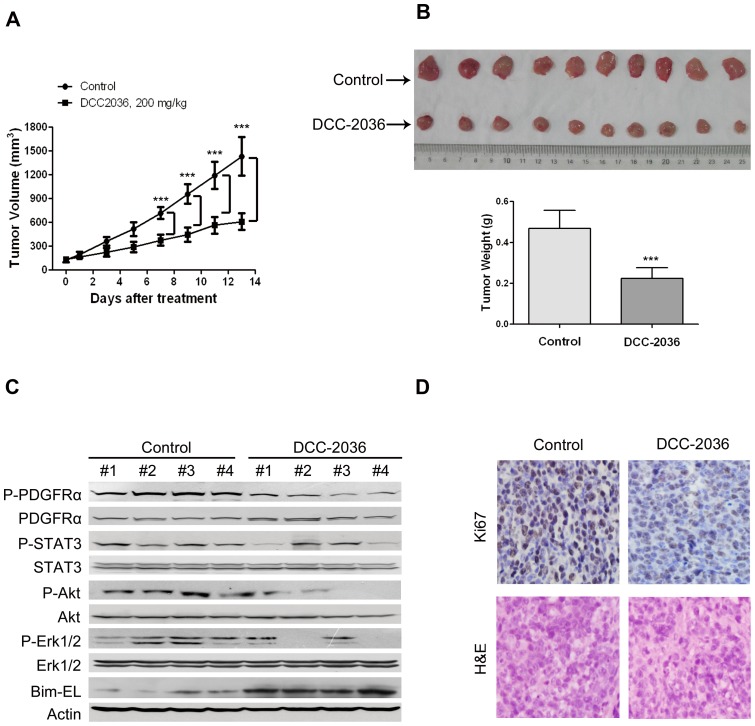
DCC-2036 abrogates the growth of xenografted T674I PDGFRα tumors transplanted in nude mice. A, the growth curves of subcutaneous xenografts of BaF3-T674I cells are shown. Nude mice bearing BaF3-T674I xenograft tumors were treated with vehicle or DCC-2036 (200 mg/kg, administered by oral gavage daily) from day 3 to 16 after inoculation of BaF3-T674I cells. The estimated tumor size was plotted versus the days after DCC-2036 treatment. Points, mean; bars, SD. Student's *t* test. B, After 13 days of DCC-2036 treatment, mice were sacrificed, tumors were dissected, weighed and photographed. Top, representative tumors from the control and experiment group are shown; bottom, comparison of tumor weights in control and experimental group. Columns, mean; bars, SD. Student's *t* test. C, Immunoblotting of the phosphorylated and total levels of indicated proteins in xenograft tissues inoculated in nude mice with vehicle or DCC-2036 treatment. D, Immunohistochemical analysis of Ki67 in xenograft tissues from mice. Hematoxylin and eosin (H&E)-stained serial sections of the same xenografts are presented.

To assess the *in vivo* effect of DCC-2036, we subcutaneously inoculated BaF3 cells expressing FIP1L1-PDGFRα T674I mutant in nude mice. When tumor xenografs were palpable (∼100 mm^3^), nude mice were randomized to receive vehicle (0.5% CMC/1% Tween 80, n = 6) or DCC-2036 (200 mg/kg in 0.5% CMC/1% Tween 80, once daily, oral gavage, n = 7) for about 2 weeks. The growth curve *vs* time of BaF3-T674I tumors was apparently inhibited by the administration of DCC-2036 compared with the control group (Figure 7A). In addition, the tumor volumes and tumor weights in the drug-treated group were remarkably lower than the control group (Figure 7B). The motor activity, feeding behavior and body weight of the control group and experiment group were all normal (data not shown).

Furthermore, Western blotting analysis of these BaF3-T674I xenograft tissues from mice exhibited downregulation of phosphorylated-PDGFRα and its downstream signaling molecules, along with up-regulation of Bim-EL (Figure 7C), which indicated DCC-2036 inhibited the activity of T674I FIP1L1-PDGFRα *in vivo*. Immunohistochemical analysis with Ki67 (to measure cell proliferation) staining showed that DCC-2036 suppressed the proliferation of BaF3 cells harboring FIP1L1-PDGFRα T674I mutant *in vivo* (Figure 7D). To conclude, all these data supported that DCC-2036 had a beneficial antineoplastic effect *in vivo* in spite of the T674I-FIP1L1-PDGFRα mutation status.

## Discussion

Targeted therapy to inhibit the oncogene FIP1L1-PDGFRα has met with some success: the first-generation tyrosine kinase inhibitor (TKI) imatinib has been reported to be effective to HES, but imatinib-resistant mutations emerged. Several mutations in the kinase domains such as D842V [32], T674I [33], S601P and L629P [34], have been demonstrated to be responsible for the drug-resistance. Of note, the “gate-keeper” mutant T674I FIP1L1-PDGFRα in HES, similar to T315I Bcr-Abl in CML, was also refractory to some second-generation TKIs, such as nilotinib, sorafenib [8]. Thus, it is really a challenge to overcome the acquired drug resistance. Combination therapy with more than one compound or other novel TKIs is advised. Imatinib combined with vinorelbine have been reported to enhance breast cancer cell growth inhibition via PDGFRβ signaling [35]. Imatinib and farnesyltransferase inhibitors combination were used to treat Bcr-Abl-positive CML cells [36]. These examples inferred that combination therapy with compounds is feasible. Meantime, the third-generation TKIs (AP24534, HG-7-85-1, DCC-2036) have been developed [10], [37], [38], but their efficacy and safety still need to be further validated in clinical trials. In this study, we mainly explored the effect of the novel TKI DCC-2036 on HES.

DCC-2036, a third-generation TKI, was rationally designed to specifically target the Bcr-Abl T315I mutant in CML by functioning as a conformational control inhibitor. The inhibition of kinase activity by DCC-2036 on unphosphorylated-Abl, phosphorylated-Abl, unphosphorylated-Abl T315I mutant and phosphorylated-Abl T315I mutant has been demonstrated (IC_50_ values were 0.75±0.11, 2±1, 5±1 and 4±1 nM, respectively) [10]. In addition, DCC-2036 also inhibited the Src family kinases (Src, Lyn, FGR and HCK) and the receptor TKs, such as KDR, FLT3 and TIE2 [10]. Notably, the inhibition of kinase activity by DCC-2036 on PDGFRα and PDGFRβ was observed (IC_50_ values were 70±10 nM and 113±10 nM, respectively) [10]. Based on the broad and notable effect of DCC-2036 on significant kinases, we evaluated the effect of DCC-2036 on WT or T674I FIP1L1-PDGFRα positive cells of HES *in vitro* and *in vivo*. Firstly, we found that DCC-2036 remarkably decreased the phosphorylation level of PDGFRα and its downstream signaling molecules such as phosphorylated-Erk1/2, phosphorylated-Akt and phosphorylated-STAT3 without any influence on the total level of these proteins. Secondly, DCC-2036 inhibited proliferation and induced apoptosis with the increased cleavage of PARP and caspase-3 in a concentration- and time-depended manner in HES cells. In addition, cytochrome *c* and AIF released from the mitochondrion into the endochylema were also increased. Data reflected that the intrinsic (mitochondrial) apoptotic pathway was involved. Flow cytometry also reflected increased apoptosis in a dose- and time-dependent manner and increased sub-G1 apoptotic population. Thirdly, DCC-2036-mediated apoptosis of FIP1L1-PDGFRα positive cells depended on the up-regulation of Bim-EL, as other apoptosis-related proteins such as XIAP, Survivin, Bax, and Mcl-1 were not apparently influenced by DCC-2036 treatment, while Bim-EL protein level was obviously elevated. Moreover, knockdown of Bim by siRNA could attenuate the DCC-2036-mediated apoptosis in HES cells. Fourthly, DCC-2036 reduced the turnover rate of Bim-EL for decelerated degradation of Bim-EL, which depended on the ubiquitin-proteasome pathway after Bim-EL was phosphorylated at Ser69 by Erk1/2. Finally, our nude mouse model demonstrated that DCC-2036 had a good anti-tumor activity *in vivo*.

To conclude, our data demonstrate that DCC-2036 has potent activity against FIP1L1-PDGFRα positive HES cells regardless of the T674 mutation status *in vivo* and *in vitro*. Moreover, inhibitory effect of DCC-2036 was comparable to sorafenib in the FIP1L1-PDGFRα T674I mutation cells. In this study, we have also shown for the first time that Bim is essential for DCC-2036-mediated apoptosis in FIP1L1-PDGFRα positive cells. Taken together, DCC-2036 may be a promising agent for treating FIP1L1-PDGFRα-positive HES which is resistant to imatinib.

## Supporting Information

Figure S1 **DCC-2036 induces apoptosis in FIP1L1-PDGFRα-expressing cells.** A, EOL-1 cells and BaF3 cells expressing WT or T674I FIP1L1-PDGFRα were cultured with DCC-2036 at indicated concentrations for 24 hours (EOL-1 cells) or 36 hours (BaF3 cells), and then cells were collected, washed, fixed and stained with Annexin V-FITC/PI (EOL-1) or Annexin V-PE/7-AAD (BaF3 cells) to detect the cell death with flow cytometry. Left, representative of three independent experiments; Right, statistical charts, One-way ANOVA with *post hoc* intergroup comparison with control by Tukey test. *P<0.05, **P<0.01, ***P<0.0001. Data are expressed as mean ± SD. C, the impact of DCC-2036 on apoptosis-related proteins. EOL-1 cells, BaF3-WT and BaF3-T674I cells were exposed to DCC-2036 at indicated concentrations for 24 hours (EOL-1 cells) or 36 hours (BaF3 cells) and then levels of apoptosis-related proteins were detected by Western blot.
(PDF)

## References

[1] Chusid MJ, Dale DC, West BC, Wolff SM (1975) The hypereosinophilic syndrome: analysis of fourteen cases with review of the literature. Medicine (Baltimore) 54: 1–27. 1090795

[2] Verstovsek S, Tefferi A, Kantarjian H, Manshouri T, Luthra R, et al (2009) Alemtuzumab therapy for hypereosinophilic syndrome and chronic eosinophilic leukemia. Clin Cancer Res 15: 368–373. 19118067 10.1158/1078-0432.CCR-08-1302 PMC4822510

[3] Vardiman JW, Thiele J, Arber DA, Brunning RD, Borowitz MJ, et al (2009) The 2008 revision of the World Health Organization (WHO) classification of myeloid neoplasms and acute leukemia: rationale and important changes. Blood 114: 937–951. 19357394 10.1182/blood-2009-03-209262

[4] Erben P, Gosenca D, Muller MC, Reinhard J, Score J, et al (2010) Screening for diverse PDGFRA or PDGFRB fusion genes is facilitated by generic quantitative reverse transcriptase polymerase chain reaction analysis. Haematologica 95: 738–744. 20107158 10.3324/haematol.2009.016345 PMC2864379

[5] Buitenhuis M, Verhagen LP, Cools J, Coffer PJ (2007) Molecular mechanisms underlying FIP1L1-PDGFRA-mediated myeloproliferation. Cancer Res 67: 3759–3766. 17440089 10.1158/0008-5472.CAN-06-4183

[6] Fukushima K, Matsumura I, Ezoe S, Tokunaga M, Yasumi M, et al (2009) FIP1L1-PDGFRalpha imposes eosinophil lineage commitment on hematopoietic stem/progenitor cells. J Biol Chem 284: 7719–7732. 19147501 10.1074/jbc.M807489200 PMC2658066

[7] Bixby D, Talpaz M (2009) Mechanisms of resistance to tyrosine kinase inhibitors in chronic myeloid leukemia and recent therapeutic strategies to overcome resistance. Hematology Am Soc Hematol Educ Program: 461–476. 10.1182/asheducation-2009.1.461 20008232

[8] Metzgeroth G, Erben P, Martin H, Mousset S, Teichmann M, et al (2012) Limited clinical activity of nilotinib and sorafenib in FIP1L1-PDGFRA positive chronic eosinophilic leukemia with imatinib-resistant T674I mutation. Leukemia 26: 162–164. 21818111 10.1038/leu.2011.181

[9] Eide CA, Adrian LT, Tyner JW, Mac Partlin M, Anderson DJ, et al (2011) The ABL switch control inhibitor DCC-2036 is active against the chronic myeloid leukemia mutant BCR-ABLT315I and exhibits a narrow resistance profile. Cancer Res 71: 3189–3195. 21505103 10.1158/0008-5472.CAN-10-3224 PMC3206627

[10] Chan WW, Wise SC, Kaufman MD, Ahn YM, Ensinger CL, et al (2011) Conformational control inhibition of the BCR-ABL1 tyrosine kinase, including the gatekeeper T315I mutant, by the switch-control inhibitor DCC-2036. Cancer Cell 19: 556–568. 21481795 10.1016/j.ccr.2011.03.003 PMC3077923

[11] Jin Y, Lu Z, Cao K, Zhu Y, Chen Q, et al (2010) The antitumor activity of homoharringtonine against human mast cells harboring the KIT D816V mutation. Mol Cancer Ther 9: 211–223. 20053766 10.1158/1535-7163.MCT-09-0468

[12] Shi X, Jin Y, Cheng C, Zhang H, Zou W, et al (2009) Triptolide inhibits Bcr-Abl transcription and induces apoptosis in STI571-resistant chronic myelogenous leukemia cells harboring T315I mutation. Clin Cancer Res 15: 1686–1697. 19240172 10.1158/1078-0432.CCR-08-2141

[13] Cools J, DeAngelo DJ, Gotlib J, Stover EH, Legare RD, et al (2003) A tyrosine kinase created by fusion of the PDGFRA and FIP1L1 genes as a therapeutic target of imatinib in idiopathic hypereosinophilic syndrome. N Engl J Med 348: 1201–1214. 12660384 10.1056/NEJMoa025217

[14] Cools J, Quentmeier H, Huntly BJ, Marynen P, Griffin JD, et al (2004) The EOL-1 cell line as an in vitro model for the study of FIP1L1-PDGFRA-positive chronic eosinophilic leukemia. Blood 103: 2802–2805. 14630792 10.1182/blood-2003-07-2479

[15] Wu Y, Chen C, Sun X, Shi X, Jin B, et al (2012) Cyclin-dependent kinase 7/9 inhibitor SNS-032 abrogates FIP1-like-1 platelet-derived growth factor receptor alpha and bcr-abl oncogene addiction in malignant hematologic cells. Clin Cancer Res 18: 1966–1978. 22447844 10.1158/1078-0432.CCR-11-1971

[16] Pan J, Xu G, Yeung SC (2001) Cytochrome c release is upstream to activation of caspase-9, caspase-8, and caspase-3 in the enhanced apoptosis of anaplastic thyroid cancer cells induced by manumycin and paclitaxel. J Clin Endocrinol Metab 86: 4731–4740. 11600533 10.1210/jcem.86.10.7860

[17] Pan J, She M, Xu ZX, Sun L, Yeung SC (2005) Farnesyltransferase inhibitors induce DNA damage via reactive oxygen species in human cancer cells. Cancer Res 65: 3671–3681. 15867362 10.1158/0008-5472.CAN-04-2744

[18] Jin Y, Chen Q, Lu Z, Chen B, Pan J (2009) Triptolide abrogates oncogene FIP1L1-PDGFRalpha addiction and induces apoptosis in hypereosinophilic syndrome. Cancer Sci 100: 2210–2217. 19671059 10.1111/j.1349-7006.2009.01283.x PMC11159907

[19] Chan CH, Li CF, Yang WL, Gao Y, Lee SW, et al (2012) The Skp2-SCF E3 ligase regulates Akt ubiquitination, glycolysis, herceptin sensitivity, and tumorigenesis. Cell 149: 1098–1111. 22632973 10.1016/j.cell.2012.02.065 PMC3586339

[20] Yang WL, Wang J, Chan CH, Lee SW, Campos AD, et al (2009) The E3 ligase TRAF6 regulates Akt ubiquitination and activation. Science 325: 1134–1138. 19713527 10.1126/science.1175065 PMC3008763

[21] Lu Z, Jin Y, Qiu L, Lai Y, Pan J (2010) Celastrol, a novel HSP90 inhibitor, depletes Bcr-Abl and induces apoptosis in imatinib-resistant chronic myelogenous leukemia cells harboring T315I mutation. Cancer Lett 290: 182–191. 19819619 10.1016/j.canlet.2009.09.006

[22] Cools J, Maertens C, Marynen P (2005) Resistance to tyrosine kinase inhibitors: calling on extra forces. Drug Resist Updat 8: 119–129. 15869901 10.1016/j.drup.2005.04.005

[23] Lierman E, Folens C, Stover EH, Mentens N, Van Miegroet H, et al (2006) Sorafenib is a potent inhibitor of FIP1L1-PDGFRalpha and the imatinib-resistant FIP1L1-PDGFRalpha T674I mutant. Blood 108: 1374–1376. 16645167 10.1182/blood-2006-02-004457 PMC1895882

[24] Goldstein JC, Munoz-Pinedo C, Ricci JE, Adams SR, Kelekar A, et al (2005) Cytochrome c is released in a single step during apoptosis. Cell Death Differ 12: 453–462. 15933725 10.1038/sj.cdd.4401596

[25] Kole AJ, Knight ER, Deshmukh M (2011) Activation of apoptosis by cytoplasmic microinjection of cytochrome c. J Vis Exp. 10.3791/2773 PMC3197050 21730954

[26] Gong Y, Somwar R, Politi K, Balak M, Chmielecki J, et al (2007) Induction of BIM is essential for apoptosis triggered by EGFR kinase inhibitors in mutant EGFR-dependent lung adenocarcinomas. PLoS Med 4: e294. 17927446 10.1371/journal.pmed.0040294 PMC2001209

[27] Gordon PM, Fisher DE (2010) Role for the proapoptotic factor BIM in mediating imatinib-induced apoptosis in a c-KIT-dependent gastrointestinal stromal tumor cell line. J Biol Chem 285: 14109–14114. 20231287 10.1074/jbc.M109.078592 PMC2863214

[28] O'Connor L, Strasser A, O'Reilly LA, Hausmann G, Adams JM, et al (1998) Bim: a novel member of the Bcl-2 family that promotes apoptosis. EMBO J 17: 384–395. 9430630 10.1093/emboj/17.2.384 PMC1170389

[29] Essafi A, Fernandez de Mattos S, Hassen YA, Soeiro I, Mufti GJ, et al (2005) Direct transcriptional regulation of Bim by FoxO3a mediates STI571-induced apoptosis in Bcr-Abl-expressing cells. Oncogene 24: 2317–2329. 15688014 10.1038/sj.onc.1208421

[30] Hubner A, Barrett T, Flavell RA, Davis RJ (2008) Multisite phosphorylation regulates Bim stability and apoptotic activity. Mol Cell 30: 415–425. 18498746 10.1016/j.molcel.2008.03.025 PMC2453504

[31] Luciano F, Jacquel A, Colosetti P, Herrant M, Cagnol S, et al (2003) Phosphorylation of Bim-EL by Erk1/2 on serine 69 promotes its degradation via the proteasome pathway and regulates its proapoptotic function. Oncogene 22: 6785–6793. 14555991 10.1038/sj.onc.1206792

[32] Lierman E, Michaux L, Beullens E, Pierre P, Marynen P, et al (2009) FIP1L1-PDGFRalpha D842V, a novel panresistant mutant, emerging after treatment of FIP1L1-PDGFRalpha T674I eosinophilic leukemia with single agent sorafenib. Leukemia 23: 845–851. 19212337 10.1038/leu.2009.2

[33] von Bubnoff N, Sandherr M, Schlimok G, Andreesen R, Peschel C, et al (2005) Myeloid blast crisis evolving during imatinib treatment of an FIP1L1-PDGFR alpha-positive chronic myeloproliferative disease with prominent eosinophilia. Leukemia 19: 286–287. 15618966 10.1038/sj.leu.2403600

[34] Salemi S, Yousefi S, Simon D, Schmid I, Moretti L, et al (2009) A novel FIP1L1-PDGFRA mutant destabilizing the inactive conformation of the kinase domain in chronic eosinophilic leukemia/hypereosinophilic syndrome. Allergy 64: 913–918. 19210352 10.1111/j.1398-9995.2009.01943.x

[35] Weigel MT, Meinhold-Heerlein I, Bauerschlag DO, Schem C, Bauer M, et al (2009) Combination of imatinib and vinorelbine enhances cell growth inhibition in breast cancer cells via PDGFR beta signalling. Cancer Lett 273: 70–79. 18809244 10.1016/j.canlet.2008.07.040

[36] Radujkovic A, Topaly J, Fruehauf S, Zeller WJ (2006) Combination treatment of imatinib-sensitive and -resistant BCR-ABL-positive CML cells with imatinib and farnesyltransferase inhibitors. Anticancer Res 26: 2169–2177. 16827161

[37] O'Hare T, Deininger MW, Eide CA, Clackson T, Druker BJ (2011) Targeting the BCR-ABL signaling pathway in therapy-resistant Philadelphia chromosome-positive leukemia. Clin Cancer Res 17: 212–221. 21098337 10.1158/1078-0432.CCR-09-3314

[38] Weisberg E, Choi HG, Ray A, Barrett R, Zhang J, et al (2010) Discovery of a small-molecule type II inhibitor of wild-type and gatekeeper mutants of BCR-ABL, PDGFRalpha, Kit, and Src kinases: novel type II inhibitor of gatekeeper mutants. Blood 115: 4206–4216. 20299508 10.1182/blood-2009-11-251751 PMC2879103

