# Supplementary figures and images for: The Conformational Control Inhibitor of Tyrosine Kinases DCC-2036 Is Effective for Imatinib-Resistant Cells Expressing T674I FIP1L1-PDGFRα
Source: PLoS One. 2013 Aug 29;8(8):e73059. doi: 10.1371/journal.pone.0073059 (PMC3756952; doi:10.1371/journal.pone.0073059)

Figure S1

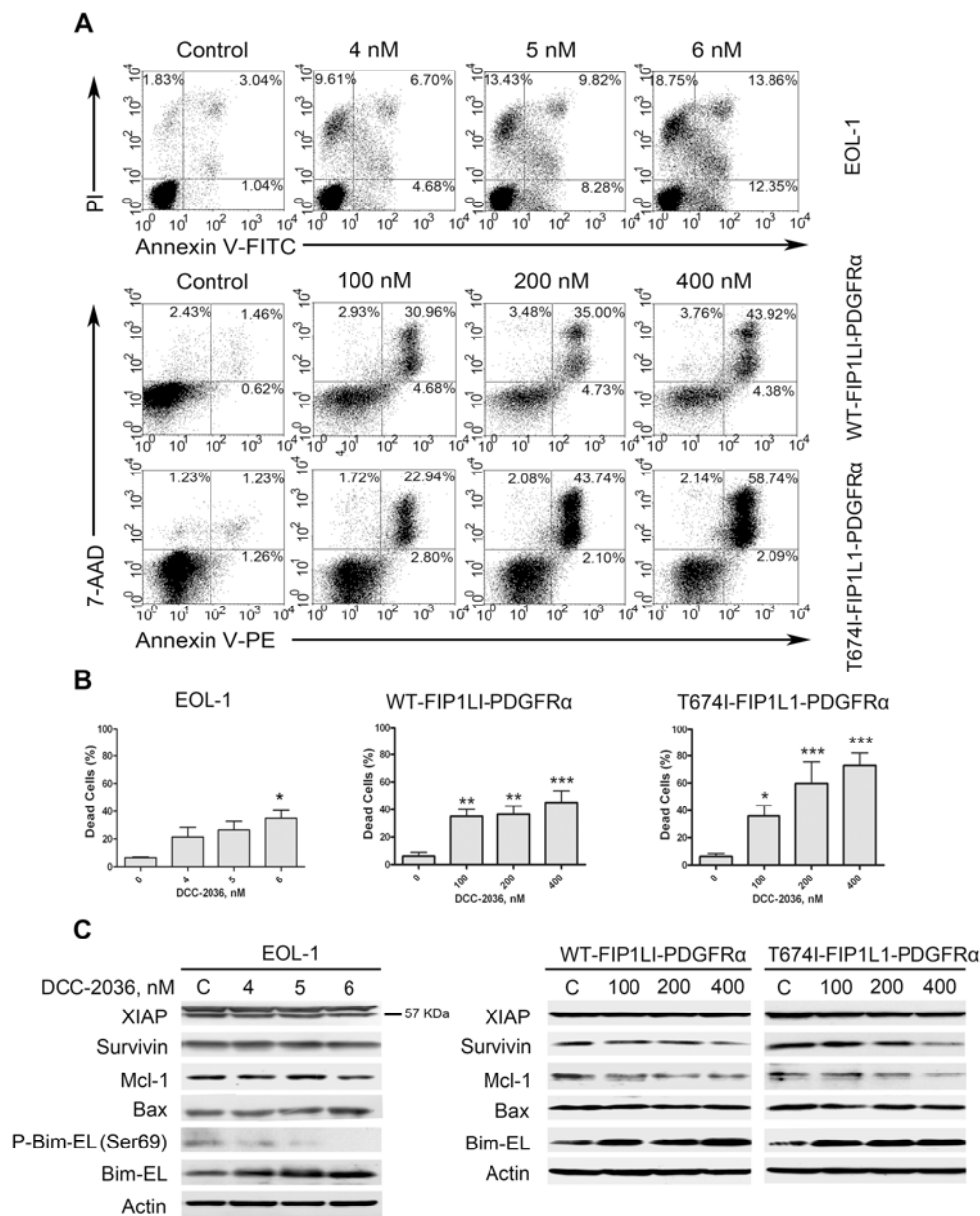

Supplement: Figure S1 — DCC-2036 induces apoptosis in FIP1L1-PDGFRα-expressing cells. A, EOL-1 cells and BaF3 cells expressing WT or T674I FIP1L1-PDGFRα were cultured with DCC-2036 at indicated concentrations for 24 hours (EOL-1 cells) or 36 hours (BaF3 cells), and then cells were collected, washed, fixed and stained with Annexin V-FITC/PI (EOL-1) or Annexin V-PE/7-AAD (BaF3 cells) to detect the cell death with flow cytometry. Left, representative of three independent experiments; Right, statistical charts, One-way ANOVA with post hoc intergroup comparison with control by Tukey test. *P<0.05, **P<0.01, ***P<0.0001. Data are expressed as mean ± SD. C, the impact of DCC-2036 on apoptosis-related proteins. EOL-1 cells, BaF3-WT and BaF3-T674I cells were exposed to DCC-2036 at indicated concentrations for 24 hours (EOL-1 cells) or 36 hours (BaF3 cells) and then levels of apoptosis-related proteins were detected by Western blot. (PDF) [file pone.0073059.s001.pdf]
